# Ecotoxicology of *Planktothrix agardhii* Cyanometabolites and Pure Microcystins: Selected Aspects of Interactions, Toxicity, and Biodegradation

**DOI:** 10.3390/toxins18010024

**Published:** 2026-01-01

**Authors:** Magdalena Toporowska

**Affiliations:** Department of Hydrobiology and Protection of Ecosystems, University of Life Sciences in Lublin, Dobrzańskiego 37, 20-262 Lublin, Poland; magdalena.toporowska@up.edu.pl; Tel.: +48-81-461-00-66 (ext. 309)

**Keywords:** cyanobacteria, non-ribosomal peptides, duckweed microbiota, biodegradation, cyanometabolites’ mixtures

## Abstract

Cyanobacterial blooms are an escalating ecological concern driven by eutrophication and climate warming. Bloom-forming cyanobacteria can produce a broad spectrum of bioactive secondary metabolites. Among these, microcystins (MCs) are the most recognised hepatotoxins; however, natural populations of *Planktothrix agardhii* also synthesise numerous non-ribosomal peptides (NRPs) with poorly understood ecological roles and combined toxic effects. This review demonstrated the role of mixtures of *P. agardhii* cyanometabolites (oligopeptides and biogenic compounds) in cyanobacterial proliferation, emphasising the rapid evolution of chemotypes. The role of *P. agardhii* oligopeptides other than MCs in the cyanobacterial toxicity to duckweeds is also discussed. Laboratory experiments indicated that crude extracts containing complex peptide mixtures may inhibit *Spirodela polyrhiza* growth more strongly than pure MC-LR, suggesting synergistic effects within natural metabolite assemblages. Particular attention is given to variant-specific degradation pathways of MCs within duckweed-associated microbiota. By integrating biochemical, ecological, and microbiological perspectives, this synthesis outlines emerging directions in the study of mixtures of cyanobacterial peptides and other compounds, microbial degraders, and macrophyte-associated bioremediation strategies aimed at mitigating cyanotoxin risks in aquatic environments.

## 1. Introduction

Cyanobacteria (blue-green algae) are prokaryotic organisms classified within the domain Bacteria. To date, 374 genera have been described, the existence of over 230 of which has been confirmed by molecular methods [1]. The increasing eutrophication of aquatic environments and climate change—particularly longer growing seasons and elevated water temperatures—are key factors promoting massive cyanobacterial proliferations and the formation of water blooms [2,3]. Although blooms in freshwater systems are typically formed by only a dozen genera and about 30 predominantly planktonic species, they represent a significant environmental issue that has been widely studied [3,4,5,6,7]. The most common bloom-forming cyanobacteria belong to the genera *Microcystis* (Chroococcales), *Aphanizomenon*, *Cuspidothrix, Dolichospermum, Raphidiopsis* (Nostocales), and *Planktothrix* (Oscillatoriales), with *Planktothrix agardhii* [6]. *P. agardhii* is a widespread, potentially toxic species capable of forming persistent (multi-month) blooms in shallow, eutrophic inland waters of temperate regions [3,8,9,10,11]. It thrives under high nutrient concentrations—especially ammonium nitrogen—enhanced turbidity, and frequent water mixing while tolerating a broad temperature range [12,13]. Projected global temperature rise and ongoing eutrophication are expected further to intensify *P. agardhii* blooms in the coming decades [3]. Such blooms contribute to the degradation of aquatic ecosystems, loss of ecosystem services, and exclusion of water bodies from use as drinking water sources, exacerbating the global water crisis [14,15].

Cyanobacteria produce a wide range of secondary metabolites, including cyanotoxins such as hepatotoxic microcystins (MCs) and nodularins (NOD), neurotoxic anatoxins (ANTX) and saxitoxins (STX), as well as numerous other bioactive oligopeptides, e.g., aeruginosins (AERs), microginins (MRGs), anabaenopeptins (APs), and cyanopeptolins (CPs) [16,17,18]. According to the CyanoMetDB database [19], cyanobacterial peptides constitute approximately 65% of all known cyanometabolites, with molecular weights ranging from 100 to 2500 Da; 57% of these metabolites are cyclic. Beyond MCs, at least 500 structurally characterized oligopeptides with molecular masses between 400 and 1900 Da have been identified [20]. Over the past two decades, increasing attention has been given to the diversity and bioactivity of metabolites produced by *P. agardhii* [8,9,17,21,22,23]. This species predominantly synthesises demethylated variants of MC-RR, including [D-Asp^3^]MC-RR and [D-Asp^3^, Dhb^7^]MC-RR, as well as [D-Asp^3^]MC-LR and [D-Asp^3^]MC-HtyR [17,24]. Additionally, *P. agardhii* produces various other nonribosomal oligopeptides (NRPs)—AERs, APs, CPLs, and MRGs—and ribosomally synthesised microviridins (MICs) [8,25,26]. The synthesis of MCs and other oligopeptides depends on complex and not fully understood environmental factors, making it impossible to predict the proportions of toxic (MC-producing) and non-toxic subpopulations within a bloom [8,9,21,22,27]. *P. agardhii* populations consist of genetically diverse strains differing in the presence and expression of genes responsible for MC and other peptide biosynthesis [9,21,26]. Consequently, they form non-clonal populations composed of several chemotypes characterized by distinct and stable peptide profiles, although environmental conditions can slightly modulate the relative abundance of specific peptides [17,28].

The number of identified secondary cyanometabolites, including those produced by *P. agardhii*, has been increasing steadily. In 2008, 80 MC variants were known [16]; by 2017, this number had risen to 240 [29], and by 2021 to over 310 [19]. In natural environments, extracellular MC concentrations can exceed 1800 µg L^−1^ [30,31]. Numerous studies have demonstrated the toxicity of purified cyanotoxins—particularly MCs—toward algae, higher plants, invertebrates (including zooplankton), and vertebrates, including mammals and humans [4,5,32,33,34,35,36]. Many cyanobacterial oligopeptides act as enzyme inhibitors [25,37,38]. The toxic effects of MC variants, such as MC-LR and MC-RR, on terrestrial plants and macrophytes [33,39,40] include alterations in cellular structure, disruption of physiological and biochemical processes (e.g., oxidative stress induction, photosynthesis inhibition), and growth retardation. However, the effects of natural mixtures of cyanobacterial metabolites containing multiple oligopeptides remain poorly understood. Most studies on cyanobacterial extracts have focused on *Microcystis* metabolites and the role of MCs while neglecting the potential influence of other bioactive oligopeptides and compounds [33,41,42]. However, recent work shows that diverse cyanopeptides strongly influence microbiota assembly during harmful blooms, highlighting their ecological significance beyond MCs [43]. Another poorly explored issue is the influence of cyanometabolites on the microbiota associated with aquatic macrophytes [44,45]. Recent studies indicate that these microbial communities are taxonomically diverse and play crucial roles in the health and functioning of both macrophytes and aquatic ecosystems [46,47]. Metagenomic analyses further demonstrate that epiphytic bacterial communities on macrophytes exhibit high functional and taxonomic diversity, enabling complex responses to environmental stressors, including cyanotoxins [48].

Despite extensive research on cyanobacterial oligopeptides [17,23,25,28,49,50,51], the ecological and physiological functions of these secondary metabolites— including those produced by *P. agardhii*—remain insufficiently understood. Although many exhibit pronounced biological activities, such as toxicity or allelopathic interactions, their specific roles within cyanobacterial populations and aquatic ecosystems remain to be elucidated [9,52,53]. For instance, Kurmayer et al. [9] demonstrated that the high frequency of genes encoding toxin and bioactive peptide synthesis in bloom-forming *P. agardhii* and *P. rubescens*, but not in non-bloom-forming *Planktothrix* species, suggests a functional link between peptide production, colonization potential, and habitat dominance—possibly through quorum-sensing mechanisms. The authors suggested that *Planktothrix* acts as an ecosystem-scale niche constructor, enhancing resource monopolization, creating positive feedback loops, and increasing persistence under stable environmental conditions. Hu and Rzymski [52] showed an auto-protective function of MCs in *Microcystis* colonies.

**Figure 1:**
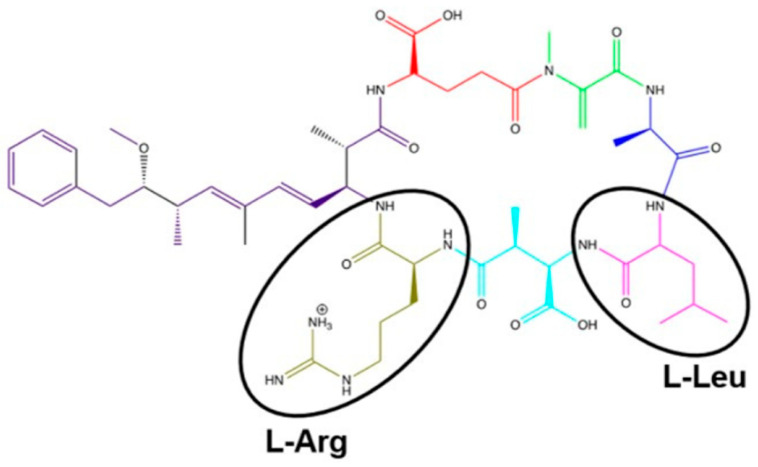
Structure of MC-LR with L-Leucine and L-Arginine. Purple indicates ADDA and red indicates D-Glu; residues that play a central role in MC toxicity, in enzyme binding, and inhibition. Green color indicates Mdha, dark blue indicates D-Ala, and light blue indicates MeAsp. Reproduced from Lad et al. [15] 2022 CC BY 4.0.

**Figure 2:**
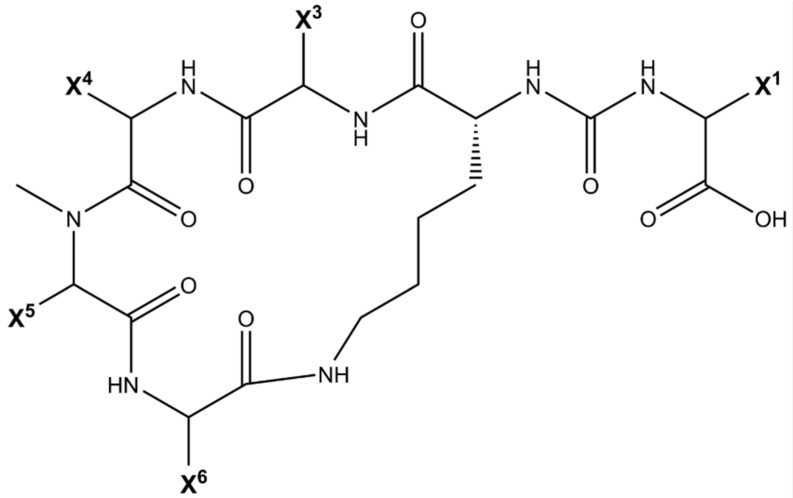
The general structure of APs. X corresponds to different amino acids in their respective positions. Reproduced from Monteiro et al. [61] 2021 CC BY 4.0.

Advancing knowledge on the toxicity, persistence, and biodegradation of cyanobacterial oligopeptide MCs remains essential. The cyclic heptapeptide structure cyclo-(D-Ala^1^–X^2^–D-MeAsp^3^–Z^4^–Adda^5^–D-Glu^6^–Mdha^7^) of MCs, the most studied and common in nature oligopeptides, determines their biological activity and toxicity [54]. This molecule contains two unique non-protein amino acids—N-methyldehydroalanine (Mdha^7^) and Adda^5^ (3-amino-9-methoxy-2,6,8-trimethyl-10-phenyldeca-4,6-dienoic acid)—as well as three D-amino acids (D-Ala^1^, D-MeAsp^3^, D-Glu^6^) and two variable L-amino acids (X^2^ and Z^4^), most commonly L-leucine and L-arginine, which define the MC variant (e.g., MC-LR, Figure 1). The toxicity of MCs is strongly influenced by their stable cyclic structure, which determines the orientation of key residues (Adda, MeAsp/Glu, Mdha) necessary for protein phosphatase inhibition (e.g., PP1, PP2A) in cells [53,54,55,56,57,58]. The presence of Adda and D-Glu residues plays a central role in enzyme binding and inhibition (Figure 1). Moreover, structural modifications—such as amino acid substitutions, methylation, halogenation, oxidation, or stereochemical changes—can substantially alter MC bioactivity and toxicity [59,60]. APs, the most common cyanobacterial oligopeptides [18,23], are cyclic hexapeptides characterized by extensive structural diversity [61] (Figure 2). They are known to inhibit several enzyme classes, including carboxypeptidases A and B, serine proteases such as chymotrypsin, trypsin, and elastase, and serine/threonine phosphatases PP1 and PP2. To date, 124 variants of anabaenopeptins have been identified across a broad spectrum of cyanobacterial taxa within the orders Nostocales, Oscillatoriales, and Synechococcales [61].

Microcystin degradation represents a key process in reducing their toxicity and removing these compounds from aquatic environments. Two major transformation pathways are recognized: abiotic (e.g., photodegradation, hydrolysis) and biotic [7]. Bacteria primarily mediate biotic degradation—currently the best-studied and most effective natural mechanism of MC removal—although fungi and plants may also contribute, albeit to a lesser extent [30]. Most MC-degrading bacteria belong to the phylum Proteobacteria and have been isolated from various aquatic environments, particularly from water columns and lake sediments [61,62,63,64,65]. These bacteria utilize MCs as an additional source of organic carbon and nitrogen [62] and possess specific gene clusters that encode MC-degrading enzymes [66]. The *mlrA–D* gene cluster encodes three hydrolytic enzymes—microcystinase (MlrA), serine peptidase (MlrB), and metallopeptidase (MlrC)—as well as a transporter protein facilitating uptake of the linearized toxin into the bacterial cytoplasm [66]. Comprehensive reviews highlight the natural occurrence, diversity and biotechnological potential of MlrA and related enzymes in microcystin degradation [67,68,69,70]. Over 20 MC degradation products have been identified, including 13 for MC-LR, one for MC-RR, and three for MC-LF, though most were reported in single studies [7].

The primary mechanism of MC biodegradation involves hydrolytic cleavage of the Adda–Arg bond, first described by Bourne et al. [66] for MC-LR. Yang et al. [67] confirmed similar degradation pathways for MC-LR ([M + H]^+^ = 995.5590) and MC-RR ([M + H]^+^ = 1038.5709). MlrA initiates the process by linearizing the cyclic molecule, followed by MlrB-mediated cleavage of the Ala–Arg or Ala–Leu bond, yielding a tetrapeptide. MlrC then hydrolyzes the bond between Adda and Glu residues, leading to complete detoxification. MlrB and MlrC can act simultaneously on different peptide bonds within linearized MCs [68,69]. Edwards et al. [70] also demonstrated that microbial consortia from natural lakes degrade MC-LF through alternative pathways involving demethylation, hydrolysis, decarboxylation, and condensation reactions, producing distinct intermediate products compared to the canonical Mlr-dependent route.

**Figure 3:**
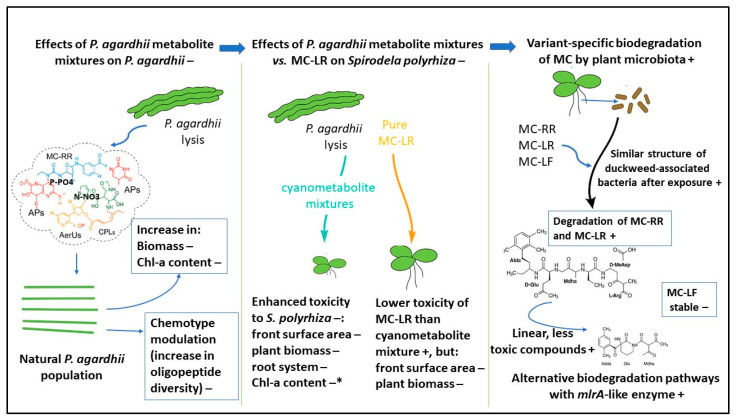
A conceptual model of the overall paper proposal with the pros (+) and cons (−) of the effects, toxicity, and biodegradation. * the effect observed only after exposure to the *P. agardhii*-dominated extract with a lower MC concentration.

The above considerations highlight significant knowledge gaps regarding the effects of *P. agardhii* metabolite mixtures—including oligopeptides other than MCs—on species proliferation, chlorophyll a (Chl-a) content, and oligopeptide composition in *P. agardhii* biomass. Moreover, little is known about the role of floating macrophytes and their associated microbiota in MC biodegradation, as well as about the impact of these toxins on the structural and functional diversity of macrophyte-associated microbial communities. To prepare this review, a comprehensive literature search was conducted in PubMed, Scopus, Web of Science, and Google Scholar, primarily covering the last 10–15 years (circa 2010–2025). Various combinations of keywords were used, including *Planktothrix agardhii*, cyanobacteria, cyanometabolites, toxicity of cyanometabolite mixtures, cyanobacterial extracts, non-ribosomal peptides, microcystins, aquatic macrophytes (e.g., duckweed), microbiota/microbiome (e.g., macrophyte microbiota), microcystin biodegradation, mlr gene, and non-mlr pathways. The search was primarily limited to English-language, peer-reviewed journal publications (research articles and reviews) to ensure quality and relevance. Studies were considered for inclusion if they examined the effects of *P. agardhii*-derived oligopeptide mixtures on aquatic plants and their associated microbiota, compared the toxicity of purified MCs versus natural cyanobacterial metabolite mixtures, or investigated microbial MC degradation pathways (including both the canonical mlr gene-cluster route and alternative non-mlr mechanisms). A conceptual model of the overall paper proposal, including the environmental pros (+) and cons (−) of effects, toxicity, and biodegradation, is shown in Figure 3.

## 2. Effect of *P. agardhii*-Dominated Metabolite Mixtures on Species Biomass, Chlorophyll a, and Oligopeptide Composition in *P. agardhii*

An essential advantage of studies on natural cyanobacterial populations over experiments with laboratory strains is the ability to capture more complex ecological interactions. Toporowska et al. [36] examined the effects of aqueous extracts derived from two cyanobacterial biomasses dominated by *P. agardhii* (95% and 82% of total biomass in Pa-A and Pa-B, respectively) collected from two eutrophic lakes in eastern Poland (Lake Syczyńskie and Lake Czarne Sosnowickie) on a natural *P. agardhii* population originating from Lake Syczyńskie. The scums contained minor shares of other cyanobacterial taxa (Pa-A: *Microcystis aeruginosa*—5%; Pa-B: *Microcystis* spp.—10%; *Aphanizomenon gracile*—8%) and differed substantially in MC concentrations—10-fold higher in Pa-A than Pa-B—as well as in oligopeptide profiles and nutrient content (P-PO_4_ and N-NO_3_ were 2–3 times higher in Pa-B). Extracts exhibited comparable Chl-a concentrations (Pa-A: 0.33–2.58 mg L^−1^; Pa-B: 0.33–2.63 mg L^−1^). Although both extracts contained a similar total number of peptides (50 and 55, respectively), Pa-A was characterized by a markedly higher AP richness (10 vs. 5 in Pa-B). In contrast, Pa-B exhibited a greater diversity of AERs (19 vs. 13 in Pa-A) and uniquely contained four MRGs and aeruginosamide—peptide classes absent from Pa-A. These qualitative differences indicate that the Pa-A bloom was dominated by chemotypes specialized in APs production. In contrast, the Pa-B bloom represented a more heterogenous assemblage with expanded biosynthetic capacities, likely influenced by the greater contribution of non-*Planktothrix* taxa. The minimal overlap between peptide profiles (Jaccard index J = 16) highlights a high degree of chemical diversification among natural *P. agardhii* populations. It underscores that bloom-derived metabolite mixtures can vary not only in toxin content but also in the structural diversity of biologically active oligopeptides.

After seven days of exposure of phytoplankton predominated (96%) by *P. agardhii* to both extracts [36], *P. agardhii* biomass and Chl-a content (per unit biomass) increased in comparison with controls (for instance, biomass rose from 188 to 558 mg L^−1^ and from 150 to 677 mg L^−1^ after exposure to extract Pa-A and Pa-B, respectively), accompanied by a slight decline in co-occurring cyanobacterial taxa, except for *Aphanothece* sp. Despite MC concentrations in the Pa-B extract being approximately 13 times higher than in Pa-A, no inhibitory effects on *P. agardhii* growth or Chl-a content were observed. Although cyanobacterial oligopeptides can stimulate blue-green algae growth [9,52], the observed biomass increase was likely driven by nutrient release from lysed cyanobacterial cells rich in organic and inorganic compounds [71], rather than by specific secondary metabolites. Notably, increasing metabolite concentrations did not alter the total MC content in *P. agardhii* biomass but affected the relative proportions of individual variants. The contribution of dmMC-LR was lowest in controls and peaked at extract concentrations of ~1.3 mg L^−1^ Chl-a. MC-RR was detected at 0.66 mg L^−1^ (Pa-A) and 2.63 mg L^−1^ (Pa-B). These results suggest that external MC concentrations did not influence intracellular MC levels, consistent with Scherer et al. [48], who found no significant effect of pure MC-LR (10–60 µg L^−1^) on *mcyB* and *mcyD* gene expression in *M. aeruginosa*. In contrast, Schatz et al. [72] observed that lysis of *Microcystis* cells, combined with MC-LR or other peptides (micropeptin, microginin), induced MC biosynthesis via an unknown autoinduction mechanism. Tonk et al. [73] also reported variation in the dmMC-RR/dmMC-LR ratio with changing light intensity, with *P. agardhii* showing higher toxicity under high light. Toporowska et al. [36] similarly found that *P. agardhii* exhibited the highest toxicity potential under optimal growth conditions, likely due to reduced light availability caused by self-shading and water discoloration by added extracts. The observed twofold reduction in dmMC-LR share at the highest extract concentrations, coinciding with biomass stagnation, may indicate a link between dmMC-LR levels and filament density. On the other hand, Zaytseva and Medvedeva [74] observed that the significant increase in the concentrations of dmMC-RR and the odor compound benzothiazole, synthesised by *P. agardhii*, corresponded to the rise in the content of nitrogen and phosphorus in the medium.

Exposure to cyanobacterial metabolite mixtures also induced significant shifts in oligopeptide composition in *P. agardhii* [36]. New peptides—such as [Asp^3^, MeSer^7^]MC-RR, MC-RR, ARED, several AERs, MRGs, CPLs, and APs—appeared, while others, mainly unidentified, disappeared. In cells exposed to Pa-A extract, AERs dominated, slightly outnumbering CPLs, MCs, and APs. In Pa-B treatments, the proportions of these peptide groups were similar. MRGs and AERs, absent in controls, represented only a minor share of total peptides in exposed cells. The highest similarity between peptide profiles was observed between *P. agardhii* exposed to both extracts (J = 0.60). At the same time, the lowest occurred when *P. agardhii* was exposed to the extracts themselves (J = 0.10–0.15), indicating no bioaccumulation of dissolved oligopeptides. Differences in oligopeptide profiles among *P. agardhii*-dominated biomasses (Pa-A, Pa-B, and control) support previous findings [17,21], suggesting the coexistence of multiple chemotypes within *P. agardhii* populations forming blooms. The relative abundance and seasonal dynamics of these chemotypes are key determinants of bloom toxicity [21,36,50]. Rapid changes in oligopeptide profiles may thus reflect shifts in chemotype composition driven by dissolved nutrients and cyanobacterial metabolites [36], or indirect effects such as differential chemotype survival and nutrient transformation by associated microbial consortia [74].

## 3. Effect of Cyanometabolite Mixtures from *P. agardhii*-Dominated Scums and Pure MCs on the Development of the Macrophyte *Spirodela polyrhiza*

Members of the family Lemnaceae, particularly *Spirodela polyrhiza* and *Lemna minor*, have recently become well-established plant–microbiota model systems [75]. Their simple morphology, rapid clonal propagation, and short doubling time make them ideal for controlled gnotobiotic experiments and for studying the transfer of microbiota between hosts. Knowledge about the effects of complex cyanobacterial metabolite mixtures—especially those containing oligopeptides other than MCs—on macrophytes remains limited [33,41,42,76,77,78]. Pawlik-Skowrońska et al. [78] conducted comprehensive experiments on young (72-h-old) *S. polyrhiza* plants exposed to pure MC-LR and to two crude extracts (Pa-A and Pa-B) obtained from cyanobacterial biomasses dominated by *P. agardhii* (Table 1). The extracts differed substantially in their oligopeptide composition: Pa-A originated from a smaller biomass (1142 mg FW L^−1^) but contained a very high MC content (12.96 μg MC mg^−1^ FW); Pa-B originated from a much larger biomass (2776 mg FW L^−1^) but had very low MC content (0.20 μg MC mg^−1^ FW). Thus, Pa-A was a high-MC chemotype, whereas Pa-B was low-MC but high-biomass. The oligopeptide composition of the two *P. agardhii*–dominated biomasses differed markedly both quantitatively and qualitatively. Extract Pa-A, derived from a lower cyanobacterial biomass, contained a total of 14 oligopeptides (4 MCs and 10 non-MC compounds), including unique AP variants such as AP-H, AP-G, and HU892. In contrast, extract Pa-B originated from a much larger biomass but exhibited very low MC levels and a richer metabolite profile comprising 17 oligopeptides (3 MCs and 14 non-MC compounds). Pa-B possessed a broader diversity of APs, additional AER variants, and uniquely contained a cyanopeptolin (planktocyclin), which was absent from Pa-A. The two extracts shared seven non-MC peptides, but ten others differed, demonstrating substantial chemotypic divergence. Previous studies had reported that crude cyanobacterial extracts containing MCs or cylindrospermopsin (Cyl) negatively affected macrophyte tissues [33]. Still, they did not differentiate between the effects of MCs and other metabolites. The experiments by Pawlik-Skowrońska et al. [78] were the first to demonstrate that defined mixtures of cyanobacterial oligopeptides can significantly influence *S. polyrhiza* growth parameters, including leaf expansion, root elongation, biomass accumulation, and Chl-a content. The toxic effects were more pronounced in leaf and root development than in chlorophyll synthesis. After 72 h of exposure, both pure MC-LR and cyanobacterial extracts significantly reduced the biomass of *S. polyrhiza* and leaf growth. Notably, the extract with 13 times lower MC content but higher oligopeptide richness (Pa-B) produced stronger phytotoxic effects, emphasizing that non-MC oligopeptides—particularly APs, AERs, and CPs—play a major role in the toxicity of *P. agardhii* blooms toward aquatic plants. Pure MC-LR—tested at concentrations up to 38 times higher than those in the extracts—had only minor effects on root and Chl-a production, confirming that mixtures of compounds other than MCs contributed substantially to the observed toxicity. These findings align with evidence that complex cyanopeptide mixtures can modulate biological responses and microbial community composition during CyanoHABs [44]. Both extracts inhibited root growth, with the Pa-B extract exerting the more substantial effect; a significant reduction in Chl-a was observed only in this treatment [78]. Similar effects of MCs and MC-containing extracts on root growth and pigment content have been reported in other vascular plants (Máthé et al., and references therein [33]). Still, these effects were not accounted for in the absence of non-MC oligopeptides. Transcriptomic data confirm that MC-LR triggers substantial changes in hormonal regulation and photosynthetic machinery in macrophytes [79]. Pawlik-Skowrońska et al. [78] showed that *P. agardhii* oligopeptides other than MCs may exhibit higher toxicity toward young *S. polyrhiza* than pure MC-LR. The peptide profile of the studied biomasses included enzyme inhibitors with confirmed cytotoxicity and inhibitory activity against proteases, protein phosphatases, and carboxypeptidases [25,37,80,81]. The phytotoxic potential of non-MC cyanobacterial oligopeptides has also been demonstrated in terrestrial plants [37], underscoring the need to include non-MC peptides in plant assays. In contrast, Gao et al. [77] found that *Myriophyllum spicatum* exposed to extracts from MC-producing and non-MC-producing *Microcystis* showed stronger responses to MC-containing extracts. However, the authors emphasized the need to consider other cyanometabolites in future studies. Similarly, Li et al. [43] demonstrated that the submerged macrophyte *Vallisneria natans* was highly sensitive to anatoxin-a (ANTX) at 0.05 μg L^−1^, and that combined exposure to MC-LR and ANTX induced antagonistic effects. Both single and combined exposures enhanced antioxidant defences, including increased activities of superoxide dismutase, peroxidase, and catalase, as well as elevated levels of glutathione, glutathione S-transferase, and malondialdehyde.

The inhibition of young *S. polyrhiza* development [78] provides mechanistic insight into the depletion of macrophyte populations frequently observed in cyanobacteria-dominated water bodies [82]. Although earlier studies reported the accumulation of MC and ANTX in aquatic plants [42,43,83], Toporowska [84] found no evidence of MC bioaccumulation after a 9-day exposure of mature *S. polyrhiza* plants to pure MC-RR, MC-LR, and MC-LF. This suggested a mechanism that could protect macrophytes against MC bioaccumulation. Such a mechanism may be linked to the structure of the associated microbiota and the presence of potential MC degraders.

**Table 1:** The effects of *P. agardhii* metabolite mixtures (extracts) vs. pure microcystins (MC-LR, MC-RR, and MC-LF) on aquatic plants and microbiota systems.

Species/ System	Concentration/ Exposure	Effect	Interpretation (Toxicity vs. Nutritional Effect)	Source
*S. polyrhiza* (young 72 h plants) exposed to crude extracts from *P. agardhii*–dominated blooms (mixtures of MCs + other NRPs)	Two extracts with distinct peptide profiles; one extract had ~13× lower MCs than the another one studied; exposure 72 h.	More potent inhibition of frond and root growth by the extract with lower MC; Chl-a reduction observed only for that extract; purified MC-LR at up to 38× higher concentration produced only minor effects on roots/Chl-a.	Mixture toxicity dominates (non-MC oligopeptides contribute substantially); MCs alone do not explain phytotoxicity.	[78]
*S. polyrhiza* (mature plants + associated microbiota) exposed to pure MC variants	Nutrient-poor medium. Initial 1000 ng mL^−1^ each of MC-RR, MC-LR, MC-LF; exposure 4–9 days.	After 9 days: MC-RR −61% (to 384 ng mL^−1^); MC-LR −21% (to 886 ng mL^−1^); MC-LF no significant decline; no accumulation of MCs or products in plant tissues.	Microbiota-mediated detoxification; variant-specific removal (RR > LR ≫ LF).	[84]
*S. polyrhiza*-associated microbiota only (no host plant) challenged with pure MC variants	Nutrient-replete medium; initial 1000 ng mL^−1^ each of MC-RR, MC-LR, MC-LF; LC-MS/MS profiling; exposure 4–9 days.	After 9 days: overall RR and LR removal ~60–70%; LF negligible; degradation products detected for MC-RR (*m*/*z* 1011, 984, 969, 877, 862, 820, 615) and MC-LR (*m*/*z* 968, 953); none for MC-LF.	Variant-specific biodegradation by the bacterial consortium; pathway similar but not identical to canonical mlr (likely alternative/low-similarity enzymes).	[85]
*S. polyrhiza* + microbiota (comparative view across the two systems)	Same MC set; comparison of nutrient-poor (with plant) vs. nutrient-replete (microbiota only) conditions; exposure 4–9 days.	Faster MC-RR decay with plants (nutrient-poor) than MC-LR (61% vs. 21% in 9 days); without plants (nutrient-replete), MC-RR and MC-LR decayed similarly; MC-LF resistant.	MC-RR’s two arginine residues likely make it a better N source, accelerating decay under N limitation; nutrient regime and host presence shape outcomes.	[84,85]

## 4. Structure of the *Spirodela polyrhiza* Microbiota After Exposure to MC-RR, MC-LR, and MC-LF

Duckweed microbiota remain incompletely characterized [45,85,86,87,88]. As emphasized in a recent review, duckweed systems enable rigorous tests of microbiota assembly and function due to clonal growth, gnotobiotic tractability, and reproducible host phenotypes [75]. Microbiota are primarily environmentally acquired and subsequently filtered by host traits and microbe–microbe interactions; host control involves secretions and frond-surface metabolites [89]. Across environments, duckweed recruits a relatively conserved taxonomic/functional core dominated by Proteobacteria, Actinobacteriota and Bacteroidota. Such patterns are consistent with observations that macrophytes host diverse, functionally specialized epiphytic microbiota capable of metabolic adaptation to chemical stressors [48]. To date, the effects of cyanobacterial metabolites on the microbiota of floating macrophytes have not been assessed; scarce studies on submerged macrophytes and terrestrial plants report consortial shifts [44,45,90]. For example, *M. aeruginosa* exudates altered the biofilms of *V. natans* more strongly than extracts did. Two ecotoxicological experiments (Table 1) examined *S. polyrhiza* microbiota after 9-day exposure to pure MC-RR, MC-LR, and MC-LF: (i) plants with their microbiota in nutrient-poor tap water [84] and (ii) microbiota alone (no host) in Steinberg medium [85]. In both cases, exposed consortia (bacteria + algae) differed markedly from controls, in which sparse, almost exclusively bacterial communities developed.

### 4.1. Community Composition

Sequencing of the microbiota-only experiment revealed high structural diversity (29 phyla; 478 genera) with dominance of *Methylophilus*, accompanied by *Chrysobacterium*, *Pseudomonas*, *Caulobacter*, *Rhizobium*, *Brevundimonas*, *Acidovorax*, *Muribaculaceae*, *Aminobacter*, and *Sediminibacterium* [85]. Proteobacteria prevailed in all samples (80.1–88.8% relative abundance), followed by Bacteroidota (7.6–15.0%) and Firmicutes (2.0–6.5%), consistent with patterns observed in *V. natans* biofilms [45] and in duckweed cores [47,88]. Verrucomicrobiota occurred at low abundance [85], in line with earlier rhizoplane isolates from *S. polyrhiza* [86]. Eukaryotes were consistently present: *Chlorella* spp. and flagellate green algae occurred in both studies; *Kirchneriella* sp. appeared only in one MC-RR control with plants [84]. The diatom *Navicula* sp. was detected only when the host was present, suggesting host-dependent recruitment.

### 4.2. Abundance Patterns and Variant-Specific Effects

Without the plant host, bacterial abundance after 9 days was ~11× higher than with plants; *Chlorella* and flagellates were ~160× and ~1000× more abundant, respectively [85]. With the host, eukaryotes (dominated by *Chlorella*) were several hundred (4500×) less abundant than bacteria, depending on the MC variant [84]. In nutrient-poor conditions with plants, MC-RR appeared most inhibitory to bacteria: mean abundance 7.9 × 10^6^ cells mL^−1^ vs. 9.3 × 10^6^ (MC-LR) and 17.5 × 10^6^ (MC-LF); only the MC-RR vs. MC-LF contrast was statistically significant [84]. This pattern was not reproduced in nutrient-replete, host-free conditions, where no significant differences among variants were detected [85]. Amplicon profiling showed similar phylum/genus-level compositions across variants; alpha-diversity indices suggested slightly less even communities with MC-LR, while beta-diversity differences were not significant. Targeted tests nonetheless resolved subtle taxon shifts:

– Phyla: higher Proteobacteria in MC-LR than MC-LF; no significant differences for MC-RR vs. MC-LR.
– Genera: MC-RR vs. MC-LR showed lower *Methylophilus* and higher *Caulobacter*, *Brevundimonas*, *Hydrogenophaga*, *Alistipes*, *Blastomonas*; MC-RR vs. MC-LF showed higher *Hydrogenophaga*, *Lachnospiraceae* NK4A136 group, *Heliimonas*, but lower Rikenellaceae RC9 intestinal group, *Ruminococcus torques* group, *Desulfovibrio*, NK4A214 group. MC-LF exceeded MC-LR in *Caulobacter*, *Brevundimonas*, *Bosea*, *Helicobacter*, *Rikenellaceae RC9*, *Ruminococcus torques*, *Desulfovibrio*, and NK4A214.

Findings align with the last report that complex cyanometabolite mixtures (beyond MCs) can depress dominant epiphytes and shift major phyla [43]. Combined ANTX + MC-LR exposures in *V. natans* elevated N-acyl-L-homoserine lactones, implicating quorum-sensing modulation [43].

### 4.3. Algal Responses

With plants present, MC-LF—least inhibitory to bacteria—most strongly suppressed *Chlorella* (1.91 × 10^3^ cells mL^−1^) and *Navicula* [84]. These effects were not reproduced in the absence of the plant host [80]. MC-RR appeared more toxic to flagellates in the host-free system, opposite to the first experiment, suggesting context dependence (nutrient status, host presence) and possible species/strain specificity [91].

### 4.4. Role of Degradation and Nutrients

Toxicity differs among MC variants (mouse assays: MC-LR/MC-LA most toxic; cell assays: MC-LW/MC-LF > MC-LR) [6,58,92], and some MC-LR biodegradation products are less toxic than the parent (PPIA; [93]). In the microbiota-only study [85], MC-LR and MC-RR degraded at similar rates. With plants and low nutrients [84], MC-RR degraded ~3× faster than MC-LR—likely reflecting its two arginine residues and its superior value as a nitrogen source. Degradation products appeared after four days in the experiment with plants and lower nutrient levels, and after nine days in the experiment without plants and higher nutrient levels, suggesting that differing nutrient regimes and host presence likely modulated microbial activity and community structure. The various MC-detection rates by numerous bacterial taxa from different environments [7,93,94,95,96,97] support this interpretation.

### 4.5. Implications

Although the metagenome was not sequenced in the first experiment [84], which limited cross-study genomic comparisons, the results underscore that MC variants, nutrient status, and host presence jointly shape the *S. polyrhiza* microbiota through direct toxicity and biodegradation dynamics. These insights may inform macrophyte- and microbiota-based strategies for water treatment targeting MC removal.

## 5. Biodegradation of MC-RR, MC-LR, and MC-LF by the *S. polyrhiza* Microbiota

A significant decrease in MC-RR and MC-LR concentrations—but not MC-LF—was observed after nine days of incubation, both with *S. polyrhiza* and its microbiota [84] and with microbiota alone [85] (Table 2). The reduction in MC-LR concentration was more pronounced in the microbiota-only system, where bacterial abundance was almost 11-fold higher than in the experiment including macrophytes, while *Chlorella* spp. and flagellates were 160- and 1000-fold more abundant, respectively.

### 5.1. Identification of Degradation Products

HPLC-PDA analyses revealed six, two, and four degradation products for MC-RR, MC-LR, and MC-LF, respectively, after exposure to *S. polyrhiza* and its microbiota [84]. In the microbiota-only system [85], HPLC-PDA and LC-MS/MS identified seven MC-RR and two MC-LR degradation products, three of which were not reported in the plant experiment [84]. Eight products were newly reported in these systems. The only previously known degradation product (tetrapeptide, *m*/*z* 614.5; Bourne et al. [66]) was detected among MC-RR metabolites. MC-LR degradation yielded linear products (*m*/*z* 968, 953), whereas MC-RR degradation produced both cyclic (*m*/*z* 1011) and linear forms (*m*/*z* 984, 969, 877, 862, 820, 614.5).

In the *S. polyrhiza* + microbiota experiment, MC-RR and MC-LR degradation products appeared after four days (D-RR, F-RR, B-LR) and increased in concentration by day nine. Although MC-LF concentrations did not decline significantly, several degradation products (A-LF, B-LF, C-LF) were detected at low levels. Their occurrence—also in controls—suggests potential abiotic transformation, possibly via demethylation, hydrolysis, or decarboxylation, as reported by Edwards et al. [70]. In the microbiota-only system [80], MC-RR and MC-LR degraded at similar rates (60–70%), whereas MC-LF degradation remained negligible. MC-RR degradation was more variable across replicates. In contrast, in the plant experiment, MC-RR degradation reached 61%, nearly three times that of MC-LR (21%) [84]. Both studies were conducted under identical temperature and light conditions, but in different media—tap water and nutrient-rich Steinberg medium. Since bacteria can use MCs as nitrogen sources, the faster degradation of MC-RR (containing two arginine residues) likely reflects its higher nitrogen content. Nutrient availability may thus modulate both the rate and extent of MC biodegradation. The observed biodegradation proceeded more slowly (over several days) than in most studies using bacteria isolated from bloom-affected environments, where complete degradation can occur within hours [89]. Nonetheless, the results confirm that MC-RR, MC-LR, and MC-LF undergo distinct biotransformation pathways [84,85] consistent with previous reports demonstrating structural- and species-dependent degradation patterns [7,97,98,99]. Interestingly, Santos et al. [100] demonstrated that *Paucibacter toxinivorans* degraded non-MC peptides (APs A/B, aerucyclamides A/D) by ~99% within seven days, while MC-LR and MC-RR showed slower reductions (≈85–90%), and that degradation of MC-LR accelerated markedly in the presence of *Microcystis* extract. Studies [7,84,85,97,98,99,100,101] suggest that cyanometabolite degradation in nature is influenced by multiple factors, and further studies on oligopeptide mixtures are required.

### 5.2. Microbial Taxa, Potential Degradation Mechanisms, and Genomic Evidence

Sequencing of *S. polyrhiza* microbiota [85] identified numerous taxa known or suspected to degrade MCs [7,96,97,98,99], including *Acidovorax*, *Acinetobacter*, *Arthrobacter*, *Bacillus*, *Brevibacillus*, *Lactobacillus*, *Lysinibacillus*, *Methylophilus*, *Pseudomonas*, *Rhizobium*, *Rhodococcus*, *Sphingomonas*, and *Stenotrophomonas*. Other genera frequently found in MC-degrading consortia—such as *Bosea*, *Brevundimonas*, *Chryseobacterium*, *Hyphomicrobium*, *Polaromonas*, *Sphingobium*, and *Variovorax*—were also present, demonstrating the occurrence of MC-degrading bacteria within macrophyte-associated microbiota of Lemnaceae. Although bacterial degradation was the primary mechanism, contributions from co-occurring algae, protists, or fungi cannot be excluded, as such synergistic biodegradation has been observed in mixed microbial systems [7,98]. The absence of detectable MCs or their metabolites in *S. polyrhiza* tissues, combined with confirmed degradation in microbiota-only systems, indicates that bacterial activity was chiefly responsible for toxin removal.

Metagenomic sequencing revealed sequences homologous to the *mlrA* gene (microcystinase) in *Methylophilus* sp., genes encoding glutathione S-transferases in *Pseudomonas* spp. and *M. methylophilus*, and *mlrC*-like proteases in *Pseudomonas* spp. [85]. However, no glutathione-conjugated MC products were detected, which may reflect rapid downstream metabolism or concentrations below LOQ. The presence of putative *mlrA*-like sequences, the detection of linear heptapeptides as degradation products, and the resistance of MC-LF to degradation suggest that the linearization of MC-LR and MC-RR was likely enzymatic, mediated by a protein with low similarity to known microcystinases. This enzyme may possess higher substrate specificity than canonical MlrA, which hydrolyses a broader range of MC variants, including more hydrophobic types (MC-LW, MC-LF) [96]. This is consistent with known variability in MlrA substrate affinity reported across bacterial taxa [68]. Collectively, these findings demonstrate that the *S. polyrhiza*-associated microbiota can degrade some MC variants via diverse enzyme-mediated pathways (Table 2). They also underscore the potential of macrophyte–microbiota systems as natural components of bioremediation strategies for cyanotoxin-contaminated waters. Other aquatic plants, including *Ceratophyllum demersum* and *Pistia stratiotes*, have been shown to effectively remediate toxin-rich waters, further supporting the role of macrophytes in cyanotoxin mitigation [102].

## 6. Conclusions

This review underscores the complex interplay between cyanobacterial secondary metabolites, aquatic plants, and their associated microbiota, highlighting both ecological impacts and opportunities for bioremediation. Lysis of *P. agardhii* cells releases a mixture of compounds that can stimulate the cyanobacterium’s own biomass growth and Chl-a production, indicating a self-promoting effect of bloom decay products. This suggests that biogenic substances (e.g., nutrients from lysed cells) play a more critical role in fostering regrowth than the oligopeptides alone. No bioaccumulation of dissolved oligopeptides in *P. agardhii* biomass was observed; instead, differences in oligopeptide profiles indicate that interactions among released metabolites and nutrients may modulate the development and dominance of specific chemotypes within the population. The qualitative and quantitative composition of oligopeptides produced by different *P. agardhii* strains limit the growth of the floating macrophyte *S. polyrhiza* and reduce Chl-a content in plant tissues. Notably, non-MC oligopeptides exerted a more potent phytotoxic effect than MCs themselves. Such metabolite-mediated suppression of macrophytes, coupled with nutrient recycling from cell lysis, likely confers a competitive advantage to cyanobacterial blooms in nutrient-rich waters. Furthermore, the duckweed’s associated microbiota can play a significant role in modulating the effects of toxins and facilitating detoxification. The structure of the duckweed microbiota depends on the host plant, nutrient availability, and MC degradation processes. Macrophyte bacterial consortia show high diversity, resistance of dominant Proteobacteria (e.g., *Methylophilus*) to MC-LR, and potential to biodegrade hydrophilic variants (MC-RR, MC-LR) but not the hydrophobic MC-LF. The observed differences in degradation pathways suggest the involvement of specific detoxication enzymes, including those with MlrA-like activity. Under natural conditions, plant-associated microbiota may mitigate the toxic effects of MCs and prevent toxin bioaccumulation by degrading some MC variants into less toxic linear forms. There is a need for further studies on the toxicity of whole metabolite mixtures, with an emphasis on oligopeptide profiles and meaning, MC variant-specific degradation pathways, and the potential of macrophyte–microbiota systems as natural components of bioremediation strategies for cyanotoxin-contaminated waters.

## Figures and Tables

**Table 2:** Comparison of MC degradation pathways. An arrow means the following steps of the MC biodegradation.

Aspect	Canonical *mlrA–B–C* Pathway	Variant-Specific Pathway (Duckweed Microbiota)
Main enzymes	MlrA → MlrB → MlrC (+ MlrD transporter)—enzymes responsible for ring cleavage and peptide shortening [66,94]	Not necessarily a complete *mlr* cluster; cooperation of microbiota and host plant; possibly alternative hydrolases [85]
Degradation sequence	1. Cyclic → 2. Linear → 3. Tetrapeptide/Adda fragment → Amino acids [94,95]	1. Release/leakage → 2. Fragmentation → 3. Further degradation—sequence may be less linear or enzyme-dependent. Eight newly described products and a tetrapeptide [85]
Intermediate products	Linear MC → Tetrapeptide → Adda fragment → Amino acids [66,94]	Liner intermediates (longer peptides, Adda derivatives), but the mechanism is poorly understood [85]
Ecological system	Isolated bacterial strains under laboratory conditions [94]	Macrophyte (*S. polyrhiza*) and its associated microbiota—a complex, plant–microbial system [85]
Applied significance	Used in bioremediation, wastewater treatment, and enzymatic studies [94,96]	Demonstrates potential for natural bioremediation in aquatic ecosystems with macrophytes [85]
Limitations	*mlr* genes often undetectable in situ in natural waters [94]	Mechanism not fully elucidated; pathway inferred from variant-specific degradation patterns [85]

## Data Availability

No new data were generated or analyzed in this study. All data presented are derived from previously published sources as part of this review.

## Abbreviations

The following abbreviations are used in this manuscript:

MCs	Microcystins
NOD	Nodularins
ANTX	Anatoxins
STX	Saxi-toxins
AERs	Aeruginosins
MRGs	Microginins
APs	Anabaenopeptins
CPs	cyanopeptolins
